# Comparison of Virtual Intersection and Occlusal Contacts between Intraoral and Laboratory Scans: An In-Vivo Study

**DOI:** 10.3390/jcm12030996

**Published:** 2023-01-28

**Authors:** Florian Beck, Stefan Lettner, Lana Zupancic Cepic, Andreas Schedle

**Affiliations:** 1 Division of Oral Surgery, University Clinic of Dentistry, Medical University of Vienna, 1090 Vienna, Austria; 2 Core Facility Hard Tissue Research and Biomaterial Research, Karl Donath Laboratory, University Clinic of Dentistry, Medical University of Vienna, 1090 Vienna, Austria; 3 Division of Prosthodontics, University Clinic of Dentistry, Medical University of Vienna, 1090 Vienna, Austria; 4 Competence Center Dental Materials, University Clinic of Dentistry, Medical University of Vienna, 1090 Vienna, Austria

**Keywords:** intersection, intraoral scanning, occlusion, digital impression, virtual cast, alignment, virtual occlusal record, intermesh penetration, CAD/CAM

## Abstract

Background. The inaccurate maxillomandibular relationship of virtual casts following alignment by the vestibular scan may result in intersection (intermesh penetration) between opposing dental arch surfaces. Intersection occurs at short interocclusal distances in the occlusal contact area (OCA) and may result in infra-occluded definitive restorations. The purpose of this clinical study was to compare initial (by the proprietary scanner software) and new alignments (by a standalone 3D software) of virtual casts regarding OCA and intersection failure. New alignments aimed to rectify intersections by refinement of occlusal contacts. Material and Methods. The virtual casts of 30 patients following digital and conventional impression-taking were analyzed, which were acquired for single implant restoration in the posterior site. Digital impressions were performed by both IOS 1 (3M True Definition) and IOS 2 (TRIOS 3), either as complete- or partial-arch scans, respectively. Mounted gypsum casts were digitized as complete-arch by a laboratory scanner (LS) in enabled and disabled mode to avoid intersection [LS (+)/LS (−)]. All virtual casts were newly aligned by a 3D software. The difference of the OCA and the area of intersection were calculated for initial and new alignments, using interocclusal distance ranges of 0–100 μm, 0–10 μm or <0 μm (=intersection). The difference of the OCA was compared using a linear mixed model. The distribution of occlusal contact points per modality and alignment was assessed independently by three observers and estimated by inter- and intraclass correlation (ICC) coefficients. Results. Virtual casts following initial alignment demonstrated intersections irrespective of the modality. The mean area of the intersection was most for IOS 2 (79.23 mm^2^), followed by IOS 1 (48.28 mm^2^), LS (−) (2.77 mm^2^), and LS (+) (2.01 mm^2^) in partial-arch scans. Complete-arch scans demonstrated an area of intersection of 70.63 mm^2^ for IOS 1 followed by 65.52 mm^2^ (IOS 2), 6.13 mm^2^ [LS (−)] and 2.76 mm^2^ [LS (+)]. Newly aligned scans showed no intersections. The overall distribution of occlusal contact points demonstrated moderate reliability (ICC 0.63). Good reliability could be observed (ICC 0.9) for LS (−) scans. Conclusions. Intersections in the area of occlusal contact points are a phenomenon restricted to virtual casts, which should be considered in CAD/CAM. Initial alignments of LS are less affected by this virtual phenomenon, and contact points may be more distinct according to their anatomic region compared to IOS. Furthermore, intersections can be rectified in a 3D software by adjustment of the maxillomandibular relationship.

## 1. Introduction

Intraoral scanners (IOS) have revolutionized conventional impression-taking in all dentistry fields, especially in prosthodontics, which facilitates the fabrication of fixed restorations in the digital workflow [1]. Time-demanding steps after conventional impression-taking can be skipped in the dental laboratory, thus reducing the total production time of, e.g., single-unit implant restorations [2,3]. In addition, there is increasing evidence of the trueness and precision of IOS devices; however, its general recommendation for clinical use needs further investigation [4,5,6,7,8,9]. Furthermore, the wear of scan bodies, which are an integral part of implant restorations in the digital workflow, has been demonstrated to negatively impact the accuracy of IOS in complete-arch scans [10]. Yet, the correct identification of occlusal contacts following the alignment of both dental arch scans by the IOS software is of particular importance for clinicians to reduce chair-side adjustments of prosthetic restorations.

The alignment of the maxillary and mandibular scan in the IOS software is performed by capturing the buccal surfaces of the anterior and/or posterior teeth in the maximum intercuspal position (MIP) [11], although an increased interocclusal space might improve the accuracy of the maxillomandibular relationship, as demonstrated in-vitro recently [12]. Afterwards, the occlusal contacts can be digitally visualized as a “virtual occlusal record” [13,14,15]. The process of aligning both jaw scans with the bite scan is based on an algorithm of the IOS software, e.g., the best-fit alignment. This best-fit algorithm has an impact on the alignment of the virtual casts and is considered superior if based on an entire/section-based dataset compared to a landmark-based alignment [16]. The alignment process may further be influenced by the accumulation of stitching errors of the images, which arise particularly in complete-arch scans as the distance increases from the origin of the IOS scan [17,18]. Similarly, a tilting effect in complete-arch scans toward the site of bite registration has been reported, affecting the location of occlusal contacts [19]. Furthermore, both the number and location of teeth of the vestibular scan influence the accuracy of the maxillomandibular relationship in-vitro [20]. Hence the aforementioned factors may influence the identification of contact points of the virtual occlusal record. However, the intersection of surfaces between opposing scans is a virtual phenomenon following alignment, which does not occur in vivo or in physical casts. It is also referred to as interocclusal perforation or intermesh penetration, and may impair occlusal contacts [19]. Surface to surface intersection is a known problem in computer-aided geometric design and computer graphics [21,22]. Thus, intersection failure denotes the penetration of the virtual lower jaw model into the opposing upper jaw model in digital dentistry. The measurement of the Euclidean distance between opposing surface meshes allows the identification of intersection failure as indicated by a negative distance. The clinical relevance of this problem evolves as intersection occurs at very close distances, i.e., at occlusal contact points, and definitive restorations would result in too-light or no occlusal contact [19,23].

Many studies have focused on the trueness and precision of IOS and LS [8,24,25,26,27], the virtual occlusal record [13,14,23] or the fabrication of prosthetic restorations within the digital workflow [9,28]; however, there is a demand for analyzing digital impressions regarding intersection failure and identification of occlusal contact points. Both are affected by interocclusal distortions, which have an impact on the magnitude of contact points of the final restoration. However, this may be resolved during CAD design or chair-side by occlusal adjustments [29].

The aim of the present clinical study was thus to compare the area of intersection and the area of occlusal contacts (OCA) between initial alignments (by the proprietary IOS/LS software) and new alignments (by a standalone 3D software). New alignments were performed to rectify collisions between virtual models. Finally, the distribution of occlusal contact points between initially and newly aligned scans was analyzed by three observers.

## 2. Materials and Methods

### 2.1. Study Population

A total of 30 patients were recruited from the University Clinic of Dentistry Vienna who sought implant therapy for tooth replacement. Patients were invited to participate in the study if they had one missing tooth in the posterior site. Patients were considered eligible according to the following inclusion criteria: (1) age ≥18 years, (2) good general health (ASA I/II), (3) single missing premolar or molar, (4) healed extraction site of ≥3 months, (5) sufficient bone quantity at implant site, (6) complete natural dentition in the opposing jaw, (7) presence of all posterior teeth in occlusion. Exclusion criteria were: (1) severe systemic diseases, (2) local radiotherapy, (3) heavy smoking (≥10 cigarettes/day), (4) pregnancy, (5) unstable occlusion/functional shift or crossbite, (6) disorders of the temporomandibular joint, (7) current orthodontic treatment, (8) pathologic periodontal or pulpal conditions.

In the present study, digital and conventional impressions of these patients were analyzed regarding intersection area, OCA and the distribution of occlusal contacts. This study has been approved by the local ethical committee of the Medical University of Vienna (EK-Nr. 1108/2015).

### 2.2. Implant Placement and Restoration

Patients were rehabilitated with bone level tapered implants (Institute Straumann AG, Basel, Switzerland) following the standard surgical protocol of the manufacturer under local anesthesia (Ultracain D-S forte, Sanofi, Vienna, Austria). Suture removal was scheduled after 7–10 days. Postoperative analgesia was controlled by ibuprofen 400 mg every 6 h. After a healing period of ≥3 months, impression-taking was performed to fabricate screw-retained zirconia crowns.

### 2.3. Initial Alignment of the Scans

Digital impressions were performed either as partial- or complete-arch with IOS 1 (3M True Definition, 3M ESPE, St. Paul, MN, USA) and IOS 2 (TRIOS^®^ 3, 3Shape, Copenhagen, Denmark). Tooth surfaces were scanned with IOS 2 following IOS 1, as the latter requires coating with a spray applicator (3M Powder Sprayer; 3M, St. Paul, MN, USA). Bite registration (vestibular scan) was conducted in the maximum intercuspal position (MIP). Scans were aligned by the proprietary IOS software and exported as STL.

Conventional impressions were performed with a polyether impression material (Impregum^TM^ Penta^TM^, 3M ESPE, Seefeld, Germany) for the implant site and an alginate material (Alginoplast, Kulzer GmbH, Hanau, Germany) for the opposing jaw. The bite was registered in MIP using a vinylpolysiloxane material (Take 1^®^ Advanced Bite Registration, Kerr, Brea, CA, USA) in an upright seated position. Gypsum-based casts were poured in dental type V stone and mounted in an articulator (Artex^®^, Amann Girrbach, Pforzheim, Germany). After that, the casts were scanned in both enabled and disabled mode [LS (+), LS (−)] to avoid intersection using a laboratory scanner (Ceramill^®^ Map 600, Amann Girrbach, Pforzheim, Germany) and aligned in the proprietary software (Ceramill^®^ Mind, Amann Girrbach, Pforzheim, Germany). The scans were exported as PLY.

### 2.4. New Alignment of the Scans

**Figure 1:**
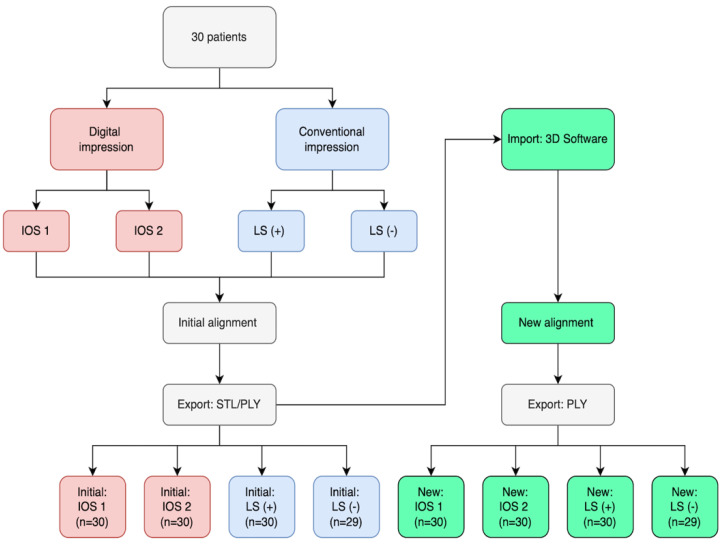
Flow diagram of initially (red, blue) and newly (green) aligned scans.

All initially aligned scans by the proprietary IOS/LS software were imported in a standalone 3D dental software (Intercusp, Rechenraum GmbH, Vienna, Austria). The upper and the lower jaw scan were automatically realigned in a newly calculated MIP impeding intersection between the occlusal surfaces (Figure 1). In order to reproduce the same newly calculated MIP, initial alignments need to fulfill the following criteria: no further displacement than ±5 mm in the occlusal plane and no more rotation than ±10° around the vertical axis from MIP. The original scan data were not modified; however, the position of the scans was changed until MIP was reached. All scans were processed successfully and exported in PLY format.

### 2.5. Measurement of Intersections and Occlusal Contacts

The nearest neighbor (signed) distances (d) between the vertices of mesh A and the triangles of the opposing mesh B were calculated to visualize occlusal contacts by a colored distance map, as previously reported [23]. The colors indicated the interocclusal distance between the scans. In order to provide the measurement of the interocclusal distances along with the export of the PLY file and for the ease of its retrieval in the statistical software R [30], the colored distance map was replaced by grey values. The distance d between two opposing mesh vertices was therefore converted to a grey value C(d) by the formula: C(d) = 255*(d-D_min)/(D_max-D_min). The interocclusal distance range was defined by a greyscale value between 0 (“D_min”) and 255 (“D_max”). Finally, encoding in the inherent RGB channel of the PLY format ensured that C(d) = R(d) = G(d) = B(d).

The intersection area as defined by a negative distance (<0 μm) and the OCA (distance range: 0–10 μm, 0–100 μm) per scan was calculated by the summation of triangular meshes of the respective grey value and expressed as mm^2^. The LS scans were split in the median sagittal plane for the partial-arch analysis according to IOS 1 and IOS 2.

**Figure 2:**
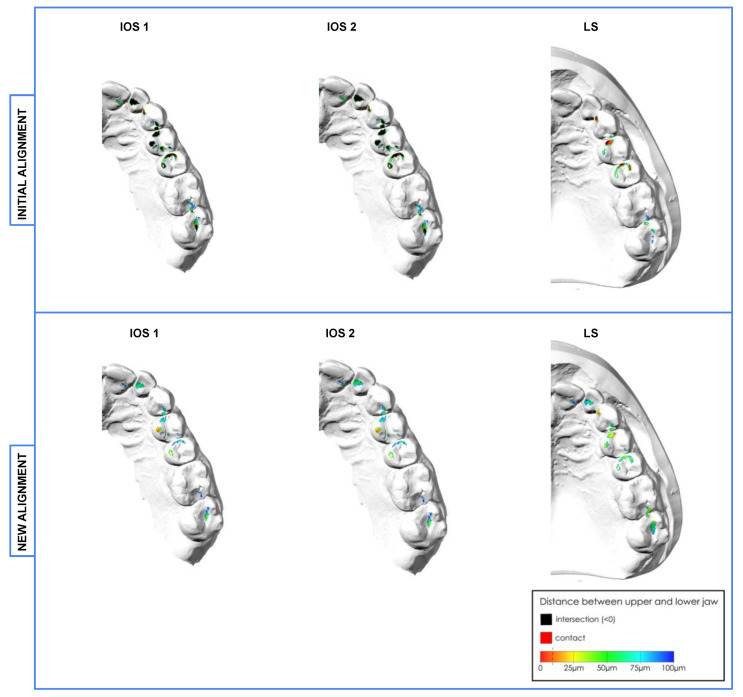
Distribution of occlusal contact points of initial and new alignments visualized by a colored distance map (interocclusal distance: max. 100 μm).

The quantification of occlusal contact points was independently assessed twice by three raters (F.B., A.S., L.Z.) according to their anatomic location [31]. Therefore, occlusal views of IOS 1, IOS 2 and LS (−) of initially and newly aligned scans were randomly depicted in a PDF (Figure 2). IOS 1/2 and type of alignment were blinded. Contact points only in the range of 10 μm were considered for the rating. Before the first assessment, a calibration session based on ten randomly chosen cases was performed to dissolve any ambiguity. The evaluation was repeated after 2–4 weeks.

### 2.6. Statistical Analysis

The PLY files were imported into R [30] using the package Rvcg [32] for the analysis of triangular meshes. Scan files were plotted to ensure that no files were changed after reading. The distance between opposing mesh vertices, intersection and the OCA were calculated from the encoded color information (grey value).

The difference of the OCA was modeled using a linear mixed model including the type of scanner [(IOS 1, IOS 2, LS (+/−)] and type of algorithm for the alignment of the scans (initial/new) as fixed factors and ID as a random factor [33].

For the number of contact points counted by several raters, we calculated inter- and intraclass correlation coefficients per scanner and algorithm and overall [34].

All computations were carried out using R version 4.2.0 [30].

## 3. Results

A total of 238 initially and newly aligned scans by IOS 1 (*n* = 60), IOS 2 (*n* = 60), LS (+) (*n* = 60) and LS (−) (*n* = 58) were analyzed in the present study. The cast model of one patient was damaged and was therefore no longer available for the disabled intersection mode scan (LS−). The mean age of the 30 patients (16 females, 14 males) was 44.6 years (range: 26–70 years). The distributions of complete- and partial-arch intraoral scans were 8 and 22, respectively, in the IOS 1 and 9 and 21, respectively, in the IOS 2 group. One of the IOS 1 scans demonstrated an incomplete mesh in one quadrant, which was excluded from the complete- and included in the partial-arch analysis.

### 3.1. Interocclusal Distances between Initially and Newly Aligned Scans

**Figure 3:**
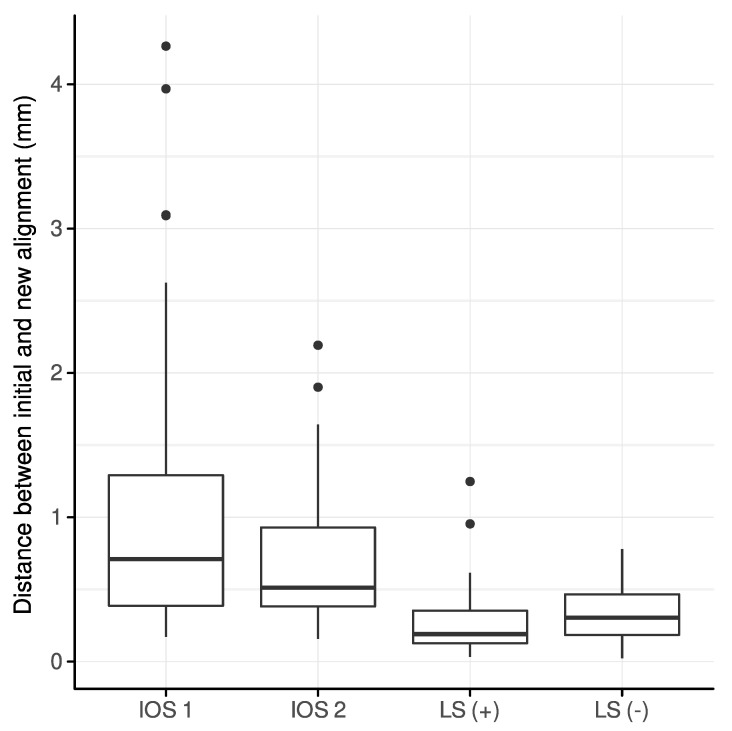
Reduction of the interocclusal distance between initially and newly aligned scans per scanner group.

The new alignment of all scans by Intercusp resulted in a reduction of the interocclusal distance, which was most distinct for both IOS 1 and IOS 2 (Figure 3). A mean reduction of 1.13 mm (range: 0.17–4.26) and 0.73 mm (range: 0.16–2.19) for IOS 1 and IOS 2, respectively, could be demonstrated. LS (+) and LS (−) indicated the least reduction of interocclusal distances between initially and newly aligned scans, i.e., a mean of 0.28 (range: 0.03–1.25) and 0.34 mm (range: 0.02–0.78), respectively.

### 3.2. Intersection

**Figure 4:**
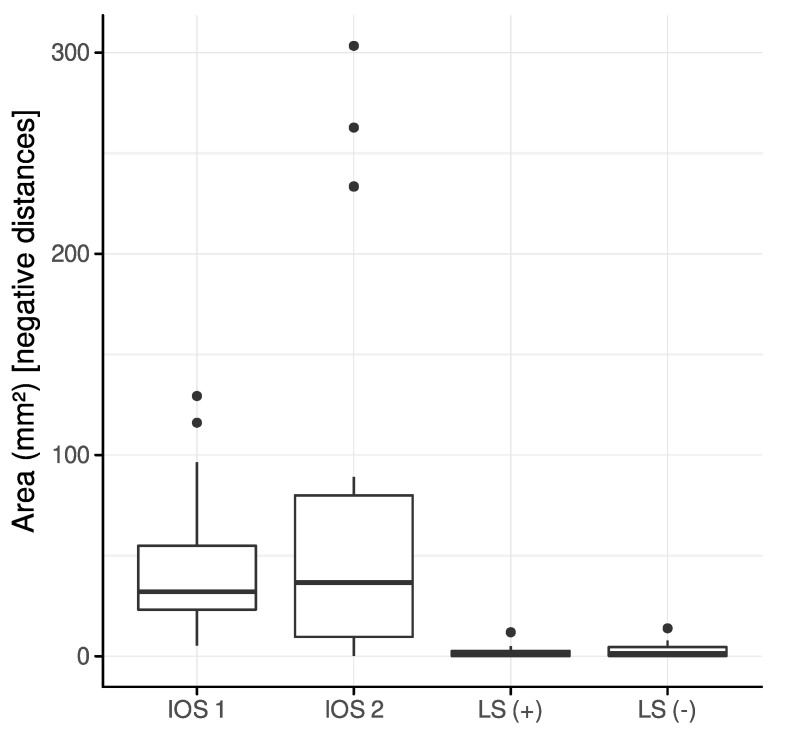
Area of intersection among initially aligned partial-arch scans.

The area of intersection, i.e., the summation of polygon areas contributing to negative distances, for partial-arch scans was largest for IOS 2 (79.23 ± 101.89 mm^2^) and IOS 1 (48.28 ± 36.74 mm^2^), followed by LS (−) (2.77 ± 3.46 mm^2^) and LS (+) (2.01 ± 2.76 mm^2^) (Figure 4).

The areas of intersection in complete-arch scans for IOS 1, IOS 2, LS (+) and LS (−) were 70.63 ± 41.13 mm^2^, 65.52 ± 82.53 mm^2^, 2.76 ± 2.71 mm^2^ and 6.13 ± 9.74 mm^2^, respectively. Newly aligned scans were free of intersection and thus did not present negative distances.

### 3.3. Difference of the Occlusal Contact Area (OCA) between Initial and New Alignment

#### 3.3.1. Interocclusal Distance Range: 0–100 μm

**Figure 5:**
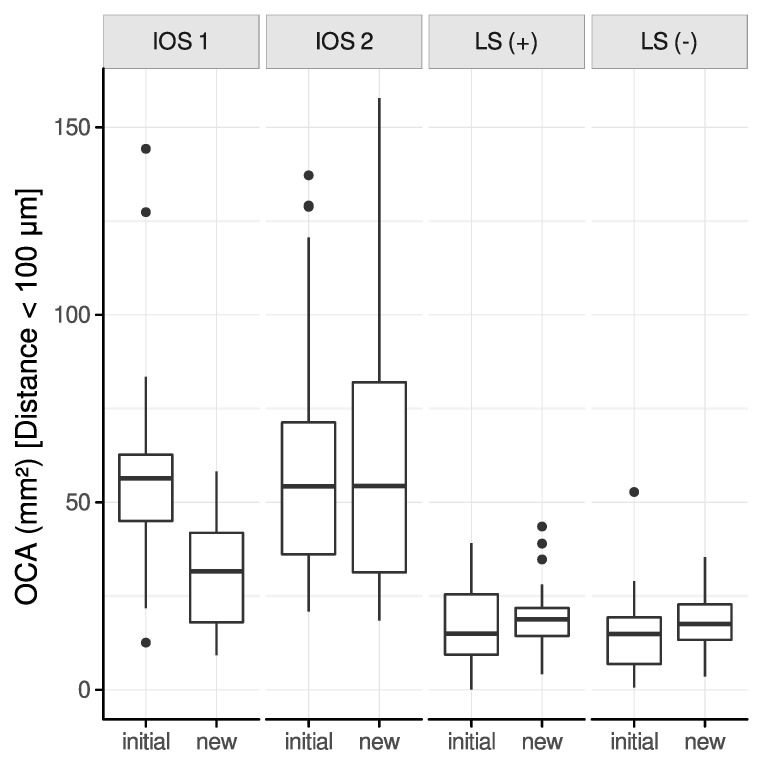
OCA between initial and new alignment of all modalities (partial-arch scans) restricted to a distance of 100 μm.

The greatest difference in reduction of the OCA between an initial and a new alignment of partial-arch scans has been observed for IOS 1 (−27.57 mm^2^), followed by IOS 2 (−2.95 mm^2^). Contrarily, both LS (+) and LS (−) indicated an increase of the OCA of 2.78 mm^2^ and 3.04 mm^2^, respectively (Figure 5). OCA was significantly reduced only for IOS 1 (*p* < 0.001).

Complete-arch scans demonstrated a similar distribution of the OCA between initial and new alignments compared to partial-arch scans. IOS 1 and IOS 2 exhibited a decrease of the OCA of −37.12 mm^2^ and −13.64 mm^2^, respectively. Again, the LS (+) and LS (−) showed an increase in the OCA, i.e., 11.53 mm^2^ and 9.12 mm^2^, respectively. IOS 1 (*p* < 0.01) and LS (+) (*p* = 0.05) demonstrated a significant reduction of the OCA.

#### 3.3.2. Interocclusal Distance Range: 0–10 μm

The maximum distance between the upper and lower jaw polygons was limited to 10 μm to simulate an occlusal contact, which should not require further adjustments in clinical practice.

**Figure 6:**
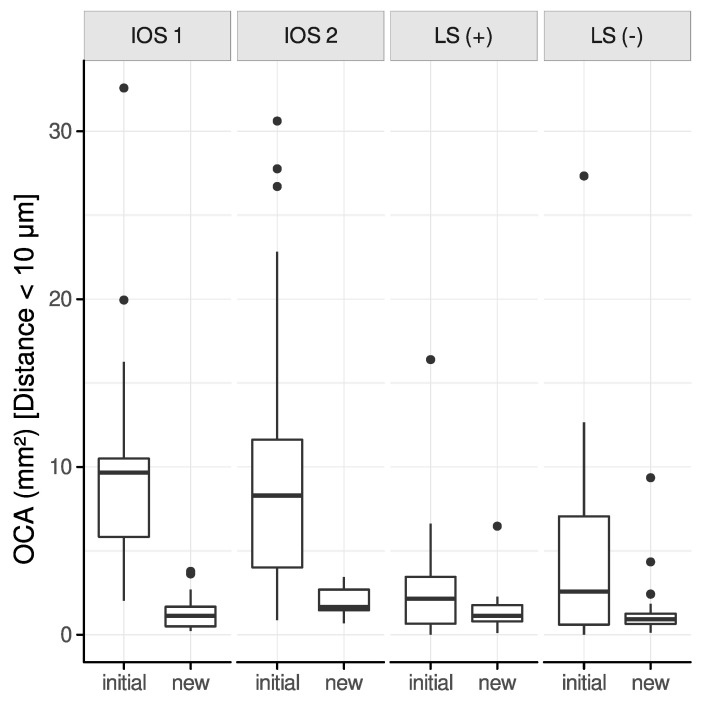
OCA between initial and new alignment of all modalities (partial-arch scans) restricted to a distance of 10 μm.

A significant decrease of the OCA for all modalities except for LS (+) between initial and new alignments could be observed for partial-arch scans (Figure 6): IOS 1 (−8.97 mm^2^, *p* < 0.01), IOS 2 (−8.88 mm^2^, *p* < 0.01), LS (+) (−1.56 mm^2^, *p* = 0.29), LS (−) (−3.39 mm^2^, *p* = 0.02).

Complete-arch scans indicated a similar distribution in terms of reduction of the OCA: IOS 1 (−10.47 mm^2^, *p* < 0.01), IOS 2 (−10.23 mm^2^, *p* < 0.01), LS (+) (−1.61 mm^2^, *p* = 0.32), LS (−) (−5.36 mm^2^, *p* < 0.01).

### 3.4. Rating of Occlusal Contact Points

The rating of occlusal contact points within the range of 0–10 μm demonstrated overall interrater reliability of 0.63 and an intrarater reliability of 0.93. The inter- and intrarater ICCs for the type of scanner (IOS 1, IOS 2, LS) were 0.38, 0.71, 0.90 and 0.94, 0.91, 0.93, respectively. Furthermore, we compared the impact of initial versus new alignments on the assessment of occlusal contact points; the interrater ICC for initially and newly aligned scans was 0.56 and 0.93, respectively, whereas the intrarater ICC was 0.82 and 0.90, respectively.

## 4. Discussion

The correct identification of occlusal contacts in the digital workflow is crucial for CAD/CAM to minimize chair-side occlusal adjustments. Studies previously reported on the trueness and precision of IOS and LS devices [6,8,26,27,35]. However, there is a demand for clinical studies analyzing and verifying virtual occlusal contacts.

The present study addressed the problem of intersection failure and further compared the OCA and contact points of the virtual occlusal record among two IOS and one LS. Intersection failure was hardly observed in the LS group in contrast to both IOS (IOS 2 > IOS 1). Newly aligned scans by the software Intercusp showed no intersection failure. The smallest OCA among all modalities was calculated for the LS group in both partial- and complete arch scans. The overall distribution of occlusal contact points (IOS 1, 2, LS—initial/new alignment) demonstrated moderate reliability (ICC 0.63). However, good reliability (ICC 0.9) could be observed if restricted to the LS group. This implies that LS scans’ contact points were more straightforwardly assigned to their anatomic location in contrast to IOS 1 and 2.

If we relate our findings to other studies investigating the virtual occlusal record, there are only a few [19,23,36] reporting on intersection failure, also referred to as (inter)mesh penetration or interocclusal perforation. This phenomenon solely linked to virtual casts occurs during the process of alignment if one virtual cast collides with its counterpart. Therefore, it can be assumed that intersections mainly happen at a very short interocclusal distance, i.e., where contact points usually evolve. Thus, in the present study, contact points were visualized by a distance map up to 100 μm and intersections by a negative distance. Furthermore, their distribution may not be even as more contact points and perforations were observed in regions closer to the vestibular scan, as reported in an in vitro study [19].

The comparison of the OCA among initially and newly aligned scans revealed a similar distribution for IOS 2, LS (+), and LS (−) if an interocclusal distance of 0–100 μm was selected. However, a noticeable reduction of the OCA could be observed for an interocclusal distance of up to 10 μm (Figure 4). The defined interocclusal distance affects the OCA, as reported in a study on masticatory performance [37]. However, the present results also suggest that refinement of contact points in newly aligned scans occurs at very short interocclusal distances.

The alignment procedure and, therefore, the presentation of occlusal contacts rely on the algorithm of the software matching the upper/lower jaw scan to the virtual interocclusal record, i.e., the vestibular scan [31]. However, varying occlusal contacts following the alignment of the same virtual cast/occlusal record have been observed depending on the software used [15]. Furthermore, stitching errors and a so-called “tilting effect” by IOS of the virtual casts towards the interocclusal record may impact the alignment and consequently distort occlusal contacts [18,19,38,39]. Quadrant scans have been propagated to overcome the tilting effect [19]. In the present study, we noticed more intersections in partial- compared to complete-arch scans, except for IOS 2. Yet, it should be noted that the sample of complete-arch scans was lower, and the distribution of occlusal contacts varies among patients. Intersections were considerably more minor in LS compared to IOS scans, which supports the theory of a “tilting effect” and errors by stitching of the images by IOS [17,19].

The implementation of a collision resolution to the algorithm of the IOS/LS software may overcome the intersection problem, as previously reported [36]. In the present study, conventional casts were scanned in LS (+) and LS (−) mode to investigate the LS software’s ability to avoid the intersection of the virtual casts. Indeed, the LS (+) group had the smallest area of intersections compared to all other modalities of initially aligned scans. However, newly aligned scans by the software Intercusp resulted in no intersections. Interestingly, the interrater agreement was considerably higher in newly compared to initially aligned scans. This may be explained by the difficulty in differentiating borderline cases, i.e., slight contact points at an interocclusal distance of 10 μm, which merge seamlessly into an intersection area. Nevertheless, the results of newly aligned scans need to be interpreted cautiously as clinical verification of the refined contact points is pending.

In the present study, the comparison of occlusal contact points between IOS and LS according to their anatomic location was revealed to be moderate overall. However, good reliability for LS scans was revealed. Yet, the interrater agreement of initial alignments demonstrated moderate reliability of 0.56. A similar agreement was observed for the comparison of IOS with an 8 μm articulating foil (AF) for the assessment of occlusal contacts (Kappa agreement: 56.1%) [40]. However, another study reported that occlusal contacts recorded by IOS were significantly less accurate and reproducible than the AF method [41]. In addition, there is still a lack of evidence regarding a universally accepted standard for identifying occlusal contacts [19,42].

This study has limitations. First, we included semi- and complete-arch scans of IOS, which may impact intersections, the OCA and the distribution of occlusal contacts. Second, we compared the distribution of occlusal contact points between two IOS and one LS; however, a clinical verification by means of AF was not performed. However, this is usually not performed within the digital workflow. Third, all patients were scanned only once in a clinical setting by two IOS devices, which does not allow conclusions regarding the scanner’s reproducibility of occlusal contacts. Finally, the rapid evolution of the IOS and LS devices’ technology may increase their trueness and precision and, in turn, impact the accurate identification of occlusal contacts. It should be noted that the trueness and precision of IOS were not investigated in this study; however, both may affect the present results.

## 5. Conclusions

Correctly identifying occlusal contact points by IOS or LS is crucial for accurate CAD/CAM restorations. Far more intersections were observed in virtual scans by IOS compared to LS. They would result in infra- or light occlusion of the definitive restoration and would therefore not be suitable for stabilizing occlusion. Resolution of collision by a standalone 3D software demonstrated no intersections in the virtual occlusal record; however, the refined occlusal contact points need to be interpreted cautiously and verified in a clinical trial.

## Data Availability

The data presented in this study are available from the corresponding author upon reasonable request.
